# A prospective multi-center study of intramedullary nailing vs casting of stable tibial shaft fractures

**DOI:** 10.1007/s10195-016-0429-4

**Published:** 2016-10-21

**Authors:** William T. Obremskey, Norele Cutrera, Christopher M. Kidd, The Southeastern Fracture Consortium

**Affiliations:** Vanderbilt University Medical Center, 1215 21st Avenue South, MCE South Tower, Suite 4200, Nashville, TN 37232 USA

**Keywords:** Tibial shaft fracture, Intramedullary nail, Casting, Non-operative treatment

## Abstract

**Background:**

The purpose of this study was to determine optimal treatment of stable tibial shaft fractures using intramedullary nailing (IMN) or casting.

**Materials and methods:**

We performed a multi-center prospective study cohort. Patients with stable tibia shaft fractures meeting Sarmiento’s criteria (isolated closed fractures with less than 12 mm of shortening and 10° of angulation) were enrolled prospectively and treated with either a reamed IMN with static interlocking screws or closed reduction followed by long-leg casting. Both groups were weight bearing following surgery. Radiographs were taken until union, and range of motion of knee and ankle joints was assessed. Malalignment (>5°) and malunion (>10°) were determined. Functional outcome measures using short musculoskeletal assessment scores (SMFA) and a knee pain score were scheduled at 6 weeks, 3 months and 6 months.

**Results:**

At 3 months, differences between the casting and IMN groups were noted in return to work (6/15 vs 3/17, *P* < 0.05); ankle dorsiflexion (7° vs 12°, *P* < 0.05); plantar flexion (28° vs 39°, *P* < 0.05); and SMFA domains of Dysfunction Index, Bother Index, daily activities, emotional status, and arm/hand function (*P* < 0.05). The SMFA mobility function demonstrated a significant trend (*P* = 0.065). At 6 months, malalignment was present in 3/15 in the casting group and in 1/17 in the IMN group (*P* = 0.02). Malunion was present in 1/15 in the cast group. One fracture in the casting group went on to nonunion and required late IMN placement at 7 months and eventually healed. There were no differences in ankle motion, SMFA scores, or return to work. There was no difference in knee pain between the groups as measured by VAS and Court-Brown pain scale at 6 months.

**Conclusions:**

Patients with stable tibia fractures treated with intramedullary nailing have improved clinical and functional outcomes at 3 months compared with those treated with casting, but there are no differences in any other outcome measure. Patients treated in a cast may have a higher incidence of malalignment or malunion.

**Level of evidence:**

Level-II prognostic.

## Introduction

Tibial shaft fractures are not an uncommon occurrence. The incidence of tibial shaft fractures is 16.9/100,000 [1]. Males have the highest frequency of fracture at 21.5/100,000, with most occurring between the ages of 10 and 20. Women have a frequency of 12.3/100,000, with the majority of fractures occurring between the ages of 30 and 40 [1]. AO type 42-A1 is the most common fracture type representing 34 % of total fractures [1].

Tibial shaft fractures have traditionally been treated with traction, casting, functional bracing, external fixation, plating and intramedullary nailing (IMN) [2]. Recently, the treatment of choice for isolated unstable closed tibia fractures has been IMN, which has shown high rates of union and low rates of malunion or rotational malalignment [3–6]. In the past, isolated, closed, stable tibia fractures have been treated with casting and functional bracing with good results [6–9]. Union of these fractures typically occurs in 16–18 weeks, and a non-union rate of 0.7 % with no post-treatment infections has been reported by Sarmiento et al. [9]. Though closed treatment has proven to be successful, a long-leg cast is difficult to manage and patients frequently experience long-term loss of joint range of motion [10].

Operative treatment has been used in patients with multiple trauma, open fractures, unstable or segmental fractures, but has been controversial in closed stable fractures [11]. Recent literature continues to demonstrate advantages of intramedullary nailing vs non-operative treatment of unstable tibial fractures [12–14]. The definitive treatment of closed tibial shaft fractures has remained somewhat debated, and a recent meta-analysis of these fractures concluded that “the published literature are inadequate for decision-making with regard to the treatment of closed fractures of the tibia [11].” It is particularly questionable whether closed, stable, minimally displaced tibial fractures are best treated with surgery or casting, and no study has been published comparing operative vs non-operative treatment of these types of fractures. This study attempts to evaluate if cast treatment or intramedullary nailing of closed, stable tibial fractures has any short or long-term advantage in healing, avoidance of complications, or functional outcome.

## Materials and methods

### Patient presentation

Institutional Review Board approval was obtained at each center prior to patient enrollment. Patients who presented to the emergency department with a closed tibial fracture were evaluated by an orthopaedic resident or attending surgeon, at which time the inclusion/exclusion criteria of the study were explained to the patient and a formal consent was obtained. Patient demographics (sex, age, mechanism of injury, type of work, and co-morbidities) and fracture type were obtained (Table 1).

**Table 1:** Patient demographics and injury

	IMN (*n* = 23)	Cast (*n* = 32)	*P* value
Age	41.9 (15.6)	43.2 (14.3)	
Sex—male	71 %	68 %	
Fracture type AO/OTA			0.89
42 A1	15	19	
42 A2	2	5	
42 A3	3	5	
42 B1	2	3	
Diabetes, *n* (%)	1 (4.4)	1 (3.1)	0.81
Smoking, *n* (%)	9 (39.1)	10 (31.3)	0.54
Anticonvulsants,* n* (%)	1 (4.4)	0 (0)	0.23
NSAIDs,* n* (%)	6 (26.1)	5 (15.6)	0.34
MOI, *n* (%)			0.41
MVA	7 (30.4)	7 (21.9)	
Pedestrian	2 (8.7)	2 (6.3)	
MCA	2 (8.7)	3 (9.4)	
Fall	12 (52.2)	20 (62.5)	

*IMN* Intramedullary nail, *NSAID* non-steroidal anti-inflammatory, *MOI* mechanism of injury, *MVA* motor vehicle accident, *MCA* motorcycle accident

### Eligibility criteria

Skeletally mature patients with isolated closed tibial shaft fractures with less than 50 % displacement, less than 10° angulation, and less than 12 mm shortening of the tibial shaft were eligible for study inclusion. Our protocol required each fracture to be at least 5 cm proximal to the tibial plafond and 5 cm distal to the tibial tubercle. Patients were also required to be competent with the English language and previously ambulatory. Exclusion criteria were open physes, multiple trauma, pathologic fractures, greater than 50 % tibial shaft displacement, open or segmental fractures, compartment syndrome, neurovascular injury, gunshot wounds, inability to have surgery secondary to existing medical problems, inability to follow-up, and unwillingness to enter the study.

### Randomization

The study was originally designed as a prospective randomized trial whereby a random number generator was used to select odd (cast) and even (IMN) numbers. The numbers and designation were placed in an opaque envelope and opened after the patient was entered into the study. Seven patients were randomized in this manner over 1.5 years. It was decided that the rate of patient accrual was not as high as anticipated, and the format of the study was changed to that of prospective cohorts. Under the new protocol, participating surgeons were asked to choose only one method of treatment with which they treated all of the participating patients under their care.

**Fig. 1:**
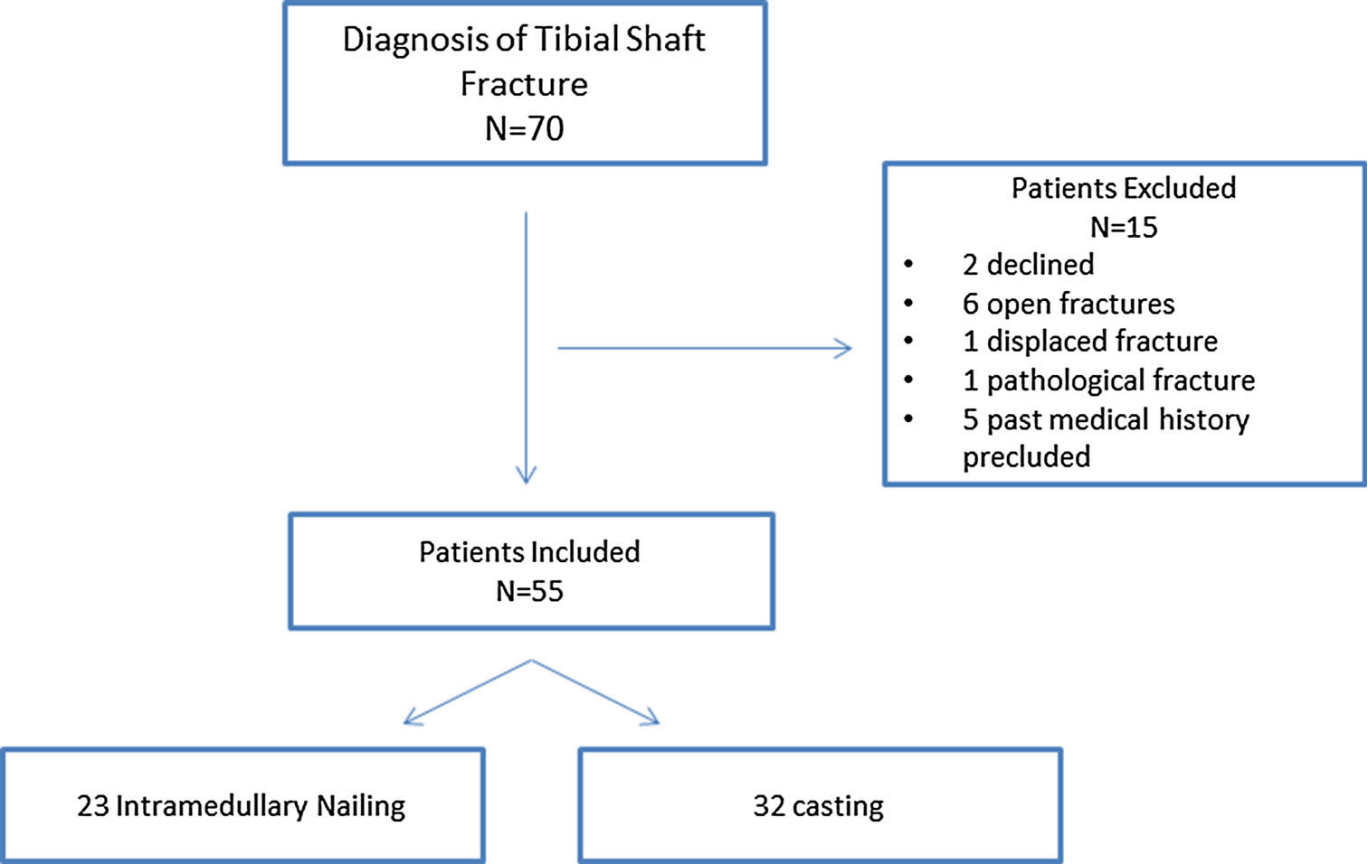
Flowchart of patient enrolment in study

Of the 70 patients evaluated for eligibility, 55 were enrolled. Of the 15 patients not enrolled, 2 declined to participate in the study and 13 met exclusion criteria (6 had open fractures, 1 had a displaced fracture, 1 had a pathological fracture, and 5 had medical issues which precluded participation). Of the remaining 55 fractures, 23 were allocated to IMN placement and 32 to treatment in a cast (Fig. 1).

### Protocol for cast treatment

Patients treated by casting were admitted to the hospital and underwent closed reduction under conscious sedation or general endotracheal anesthesia. Following reduction, each patient was placed in a standard long-leg cast with slight (5°–15°) knee flexion to allow for post-treatment weight bearing. Discharge occurred once adequate pain control was achieved and the risk of compartment syndrome was past. Patients were instructed to weight-bear as tolerated on the injured extremity. The use of assist devices, such as crutches or walker, was provided to aid in ambulation. Patients were followed weekly on an outpatient basis for anteroposterior (AP) and lateral radiographs to ensure maintenance of alignment for 2 weeks. When patients were able to comfortably weight-bear in a long-leg cast, the cast was changed to either a below-knee cast or patellar tendon-bearing cast. All patients were instructed to ambulate and continue to weight-bear as tolerated. Follow-up continued on a monthly basis until determination of fracture union, defined in this study as painless full weight bearing and radiographic evidence of bridging callus on AP and lateral views. If patients failed closed reduction during the casting period (defined as >5° of varus/valgus in any plane or shortening greater than 1.2 cm) or not healed at 6 months; these patients were subsequently treated operatively but were followed within the casting group as an “intent to treat” group.

### Protocol for IMN

Patients treated with IMN were admitted to the hospital, where they received preoperative antibiotics (Cefazolin 1 g IV) preoperatively, and every 8 h for 24 h postoperatively. Patients with a documented penicillin allergy received Clindamycin 600 mg IV every 8 h for 24 h. Skin incisions were centered over the patellar tendon, and a lateral parapatellar, medial parapatellar, or patellar tendon-splitting approach was used, based on individual patient anatomy and surgeon preference. Patients underwent closed reamed intramedullary nailing with static interlocking screws. The intramedullary canal was reamed to 1 mm greater diameter than the diameter at which cortical chatter was encountered through the isthmus. All reaming was conducted without tourniquet. Intramedullary implants were sized 1 mm smaller than the reamed diameter; no patient had a fracture gap greater than 5 mm. All implants were statically interlocked with percutaneous screws above and below the fracture site with two screws each proximally and distally. A soft dressing was placed at the end of the procedure. Patients were allowed to weight bear as tolerated with assist devices as needed. Patients were discharged once adequate pain control was achieved and the risk of compartment syndrome was eliminated. Follow-up was conducted at 2 weeks postoperatively and monthly until fracture union. Patients in both groups were asked to follow up at 3, 6 and 12 months for clinical and functional outcome assessment.

### Functional assessment

A short musculoskeletal functional assessment (SMFA) questionnaire [15] was completed at the time of admission as a baseline score of pre-injury function and thereafter at 6 weeks, 3 months, and 6 months post injury. Time to return to work was evaluated for patients with labor and non-labor employment.

### Clinical examination

Ankle and knee range of motion (ROM) was assessed at 3 and 6 months post injury. Time to successful full weight bearing was also noted. Clinical complications including loss of reduction, malunion (defined as greater than 5° of angulation in any plane or greater than 1.2 cm of shortening), infection, delayed union (greater than 24 weeks), or non-union (greater than 36 weeks) and hardware failure were noted and recorded for patients in both treatment groups. Knee pain scores were obtained at 3 months, 6 months and >24 months with a VAS score, a knee pain score by Court-Brown, and a knee function score utilized in the SPRINT study of 1300 tibia fractures [16].

### Knee pain

Patients were asked several questions regarding functional status including return to regular exercise, training and normal sporting activity. Patients also completed the Iowa knee score, which asks how a patient describes knee pain as: no pain, mild, moderate or severe. Patients also scored knee pain on a 0–10 scale (0 = none and 10 = severe) with activities of rest, kneeling, squatting, walking, and stair climbing.

### Duration of follow-up

Patients were followed until fracture union, and routine follow-ups occurred at 6 weeks, 3 months and 6 months post injury. Patients were then contacted at greater than 24 months from injury to assess long-term knee pain.

Multiple patients were lost to follow-up: 5 from the IMN cohort and 17 from the casting cohort. All of these patients failed to present for their scheduled clinic appointments at 6 weeks, 3 months, or 6 months postoperatively. Patients who were lost to follow-up at the 6-month visit but who had participated at the 3-month visit were included in the 3-month postoperative analysis. Finally, 17 patients from the IMN cohort and 15 from the casting cohort were ultimately included in the final 12-month postoperative analysis.

### Statistical analysis

An analysis of variance was performed on patient demographic data (presence of diabetes mellitus, smoking, anticonvulsant use, steroid use, NSAID use, and method of injury) to assure no confounding variables were present in the patient populations. A Chi squared analysis was conducted on infection and malunion rates with *P* = 0.05 considered significant. Clinical data of time to weight bearing, time to union, time to return to work and ROM of the knee/ankle were analyzed with a two-tailed Student’s *t* test. A two-tailed Student’s *t* test was also performed on the functional outcomes. Total and subgroup SMFA scores were compared between each group at 3 months and 6 months.

## Results

There was no significant difference found between the two cohorts in the confounding variables of diabetes (*P* = 0.81), smoking (*P* = 0.54), anticonvulsant use (*P* = 0.23), or NSAID use (*P* = 0.34) (Table 1).

Of patients with 6 month follow up, AO/OTA Fracture classification is 42A1-18, 42A2-6, 42A3-4, 42B1-4. No differences were seen between each group.

### Range of motion, alignment, and union

At the 3-month time point, the mean ankle dorsiflexion and plantar flexion in the casting cohort were 7.4° and 27.5°, respectively, compared to 12.4° and 38.6°, respectively, in the IMN cohort. These values demonstrated a significant difference with *P* = 0.012 and *P* = 0.027, respectively. At 6 months, the casting cohort demonstrated dorsiflexion and plantar flexion of 12.3° and 36.7°, while the IMN cohort demonstrated values of 15.0° and 33.4°, with *P* = 0.259 for dorsiflexion and *P* = 0.943 for plantar flexion. No differences were noted at 12 months (Table 2).

**Table 2:** Ankle range-of-motion

	IMN	Cast	*P* value
3 months			
Dorsiflxion	27.5	7.4	0.012
Plantarflexion	38.6	12.4	0.027
6 months			
Dorsiflexion, degrees	15.0	12.3	0.26
Plantar flexion, degrees	33.4	36.7	0.94
12 months			
Dorsiflexion, degrees	13.2	12.3	0.66
Plantar flexion, degrees	38.6	39.2	0.91

At the 6-month time period, 3/15 of fractures treated with casting and 1/17 of patients treated with nailing were judged to be malaligned (angular deformity 6°–10° in any plane) (*P* = 0.02). Malunion (alignment >10° in any plane) was present in 1/15 fractures in the casting cohort. No other complications were seen in either group beyond 6 months.

**Fig. 2:**
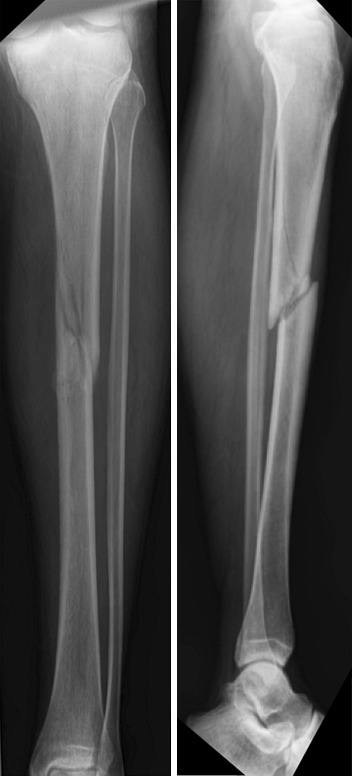
Anteroposterior (AP) and lateral view of a fracture in the casting group that went on to nonunion and required late intramedullary nail (IMN) placement at 7 months and eventually healed

At 3 months, radiographic union was demonstrated in 4/15 of fractures treated with casting and 9/17 of fractures treated with IMN (*P* = 0.2231). At 6 months, union had improved in both groups to 12/15 and 16/17, respectively (*P* = 0.3192). Only one fracture in the casting group went on to nonunion and required late IMN placement at 7 months and eventually healed (Fig. 2).

### Short musculoskeletal functional assessment scores

At 3 months, there were significant differences between the casting and IMN cohorts in the SMFA domains of Dysfunction Index (38 vs 16, *P* = 0.008), Bother Index (51 vs 18, *P* = 0.023), daily activities (58 vs 18, *P* = 0.0093), emotional status (44 vs 19, *P* = 0.0147), and arm/hand function (5 vs 1, *P* = 0.0536). Scores for the Mobility Function domain were 45 for casting and 28 for IMN, *P* = 0.0623.

At 6 months, there were no significant differences in any of the SMFA domains of Dysfunction Index (25 vs 19, *P* = 1), Bother Index (27 vs 19, *P* = 0.8571), daily activities (35 vs 21, *P* = 1), emotional status (26 vs 21, *P* = 0.881), arm/hand function (8 vs 1, *P* = 1), or mobility function (33 vs 36, *P* = 0.881). No differences were seen at 12 months as well in any domain or Index (Table 3).

**Table 3:** Short musculoskeletal functional assessment domains

	IMN	Cast	*P* value
6 months			
Dysfunction index	25	19	1.00
Bother index	27	19	0.86
Daily activities	35	21	1.00
Emotional status	26	21	0.88
Arm/hand function	8	1	1.00
Mobility	33	36	0.88
12 months			
Dysfunction index	20	19	0.83
Bother index	56	20	0.32
Daily activities	21	22	0.89
Emotional status	25	23	0.70
Arm/hand function	2	2	0.93
Mobility	34	29	0.56

### Return to work

At 3 months, 6/15 of patients treated with casting and 13/17 of those treated with an IMN had returned to work; this difference was significant with *P* = 0.04 (Table 4). At 6 months, 12/15 of patients treated with casting and 17/17 of those treated with IMN had returned to work (*P* = 0.48).

**Table 4:** Return to work and pain scores

Return to Work	IMN	Cast	*P* value
3 months	13/17 (76.5 %)	6/15 (40 %)	0.04
6 months	17/17 (100 %)	12/15 (80 %)	0.48
Pain			
Iowa knee score pain >24 months			0.059
No pain	5/15	5/17	
Mild	9/15	6/17	
Moderate	0/15	3/17	
Severe	1/15	3/17	
Pain (VAS 0–10)			
Rest	1.0	2.3	0.51
Kneeling	3.2	3.3	0.65
Squatting	3.8	4.7	0.98
Walking	1.8	2.7	0.60
Stair climbing	1.9	5.3	0.24

### Activity and knee pain

At 6 months patients had no differences in the response to return to regular exercise, training or normal sporting activity (*P* = 0.58; *P* = 1.00; *P* = 0.15, respectively). At >24 months, Iowa knee score description of knee pain in the cast vs IMN groups was: no pain (5/15 vs 5/17), mild (9/15 vs 6/17), moderate (0/15 vs 3/17) or severe (2/15 vs 2/17). (Fischer’s exact test, *P* = 0.59). Patients scores of knee pain on a 0–10 scale (0 = none and 10 = severe) were no different with any activity with the exact Wilcoxon rank sum test: rest (*P* = 0.51), kneeling (*P* = 0.65), squatting (*P* = 0.98), walking (*P* = 0.60), stair climbing (*P* = 0.24) (Table 4).

## Discussion

It is frequently reported that closed fractures of the tibial shaft are among the most common long-bone fractures encountered in modern orthopedic practice [1]. Interlocked intramedullary nailing [5] and closed reduction with long-leg casting and subsequent functional bracing [9, 17, 18] are generally favored over open reduction and plating [19] for fixation, although a meta-analysis by Littenberg et al. [11] was unable to determine the optimal method of stabilization among any of these three methods. The authors cited a lack of published data regarding treatment of closed, stable tibial shaft fractures as the reason for the absence of an evidence-based solution.

Sarmiento et al. [20] have consistently reported good results treating closed tibial shaft fractures using conservative management, and reported 450 closed tibial fractures treated with functional bracing; of these fractures, 90.0 % healed with less than 8° angulation in frontal or sagittal planes, 94.2 % healed with less than 12 mm shortening, and only 0.9 % resulted in nonunion. These results were similar to an earlier study evaluating 780 tibial shaft fractures, a mixture of closed injuries and open fractures with only minor soft tissue injury. Of these, 90.0 % healed with 10 mm of shortening or less, 75 % with 5° angulation or less in any plane, and 2.5 % failed union [9].

In a similar fashion, many authors have addressed the success of intramedullary nailing in treating tibial shaft fractures [5, 6], though most frequently in the context of comminution, a large degree of angulation or displacement, or an open fracture pattern. Placement of an IMN carries with it the inherent risks of infection, postoperative compartment syndrome, chronic knee pain, and anesthesia-related risks [14, 17], while the risks of conservative management are largely related to malunion or nonunion [5].

There are certain variables that increase the likelihood of adverse events. The Sprint study (Study to Prospectively Evaluate Reamed Intramedullary Nails in Patients with Tibial Fractures) found that there was in increased risk of negative events (unplanned intervention of hardware failure) in patients that suffered the injury due to high energy trauma [OR] = 1.57; 95 % confidence interval [CI], 1.05–2.35) and a fracture gap (OR = 2.40; 95 % CI, 1.47–3.94), and full weight-bearing status after surgery (OR = 1.63; 95 % CI, 1.00–2.64). This study mirrored our findings in regards to smoking and the use of NSAIDs postoperatively. There was no increased risk with the use of nonsteroidal anti-inflammatory agents or smoking status [21].

There have been relatively few studies comparing casting and intramedullary nailing for the treatment of tibial shaft fractures. A prospective randomized trial by Hooper et al. [3] and two cohort studies by Bone et al. [4] and Alho et al. [5] all indicated better functional and clinical outcomes in patients treated with intramedullary nailing over those treated conservatively, but all three studies included large numbers of fractures that were open, segmented, comminuted, or extremely displaced or angulated.

In a retrospective review of prospectively collected data comparing casting and IMN in regards to outcome, Batta et al. [22] found that, although IMN had a slightly shorter time to union, lesser time off of work, fewer outpatients visits, less leg length discrepancy, less anterior posterior angulation and less varus valgus angulation, there was no statistical functional difference between IMN and casting at an average of 4.3 year follow up. This suggests that although IMN does shorten recovery, there is no difference in regards to overall outcome after 4 years.

This study aims to begin filling the obvious gap in the clinical literature by directly comparing alignment, time to union, return to work and functional outcome in stable, closed tibial shaft fractures treated by either IMN placement or closed reduction with casting.

The results of this study were unable to identify that a clear difference exists between patients treated with intramedullary nailing and those treated with casting and functional bracing at 6 months postoperatively or later. Ankle mobility was essentially equal between the two cohorts, and similar numbers of patients had undergone radiograph-evaluated fracture healing and returned to work. Interestingly, this outcome represented a resolution of multiple disparities between the two cohorts that had existed at the 3-month time point. At that time, patients treated with intramedullary nailing were better able to dorsiflex and plantar flex their ankles, half had experienced radiographic healing, and more than 75 % had returned to work, compared to 40 % of the casting cohort. At 3 months, IMN patients scored significantly better on all but one domain of the short musculoskeletal functional assessment, suggesting that return of functionality and quality of life progressed much faster in this cohort.

Our data on function is similar to previously published data. A prospective cohort study, looking at the functional outcomes after IMN, found that the mean normalized SF-36 scores (physical composite score—PCS 48.9, mental composite score—MCS 51.8) and the mean normalized short musculoskeletal functional assessment scores (50.7) (Bothersome Index, Functional Index) were not statistically different (*P* = 0.05) from the reference population norms after a mean 14 year follow up [23].

Of some concern is the fact that malalignment was present in 3/15 and malunion in 1/15 patients treated with casting, compared to malalignment in 1/17 and malunion in 0/17 patients treated with an IMN. While this study did not have sufficient power to establish a significant difference between the two cohorts, these results may suggest that casting predisposes patients with closed, stable tibial shaft fractures to malalignment or malunion. One question that needs to be answered is whether or not malunion increases the incidence of clinical osteoarthritis. The evidence is variable in the literature. In a study by Milner et al. [24], 164 patients were evaluated at an average of 36 years. With this study comparing the clinical signs of osteoarthritis of the injured leg and the contralateral leg, it found that the injured leg had higher rates of pain of the knee with passive range of motion 12 (7.9 %) vs. 5.6 (4 %), ankle pain with passive range of motion 13 (9.0 %) vs. 3 (2.1 %), objective ankle stiffness 10 (6.9 %) vs. 2 (1.4 %), subtalar pain with passive range of motion 13 (9.0 %) 2 vs. (1.4 %) and subjective subtalar stiffness 35 (24.1 %) 5 (3.4 %). Although there was significant differences in pain and stiffness in the injured and uninjured legs, using Mann-Whitney statistical analysis, it was discovered that there was no statistically significant association between malunion and subjective and clinical evidence of osteoarthritis.

The most common complication 1 year after treating a tibia shaft fracture with an IMN is knee pain, which has been reported in up to 40 % of patients [16, 25]. Knee pain can persist at long-term follow-up. Connelly et al. [26] found that 22 % of patients had persistent knee 22 years after surgery. In a review article, Katsoulis et al. [27] reviewed 11 retrospective studies and nine prospective studies to assess the incidence and predictors of anterior knee pain after tibia nail implantation for a tibia shaft fracture. A total of 1460 patients was evaluated and the mean incidence of anterior knee pain was 47.4 % ranging from 10 % to 86 %. Nail removal was reported in eight studies and knee pain either persisted or was relieved after nail removal. Findings from this review indicated that anterior knee pain was most closely associated with a transtendinous approach and prominent nail. Looking at 56 patients after treatment of IMN, LeFaivre et al. [28] found that 15 (26.7 %) denied any knee pain with activity, while 41 (73.2 %) had at least moderate knee pain with activity. Interestingly, 25 of the 41 patients with knee paint stated that this pain did not limit activities.

In the present comparative trial, the incidence of knee pain as judged by a knee function questionnaire was no different in either group at 6 months or >24 months. Knee function scores were also no different at 6 months or 24 months following injury.

This investigation evaluates a common, well-defined injury with a consistent presentation and clear outcome goals. It was weakened, however, by the presence of lower-than-expected patient accrual, a high rate of patients lost to follow-up, and a change in the format from prospective randomization to prospective cohort assignment. The inherent problems of a randomized format (namely, expense, complexity, and patient and surgeon willingness to participate) proved infeasible, and it was felt that a better study could be produced using a cohort method. Nonrandomized cohort studies have been shown to produce similar results to randomized studies on the same topic, especially when they are prospective studies and outcomes evaluators have been blinded and outcomes are objective [29]. We had a significant lost to follow up in the cast 53 % (17/32) vs the IMN 26 % (6/23). This discrepancy could bias results in either direction. In general patients with a persistent problem return for follow up. Late complication in patients treated non-operatively is less likely and improved follow up in non-operatively treated injuries would more likely than not improve overall results of the cast treatment group.

In conclusion, this study evaluated stable tibia shaft fractures treated by casting or IMN. Patients with a stable tibia shaft fracture may have equal long-term results with a cast or an IMN, but may be able to return to work earlier with higher functional scores and with more reliable alignment following IMN.
